# T127. OFFSPRING OF ANTENATALLY DEPRESSED MOTHERS AND PARENTS WITH SEVERE MENTAL DISORDER – A LONG FOLLOW-UP IN THE NORTHERN FINLAND 1966 BIRTH COHORT

**DOI:** 10.1093/schbul/sby016.403

**Published:** 2018-04-01

**Authors:** Pirjo Mäki, Tiina Taka-Eilola, Graham Murray, Juha Veijola

**Affiliations:** 1 University of Oulu, Oulu University Hospital; 2 University of Oulu; 3 University of Cambridge

## Abstract

**Background:**

Depression during pregnancy is common, but long-term outcomes in the offspring of antenatally depressed mothers are unknown. Among severe mental disorders at least schizophrenia is considered to be a neurodevelopmental disorder acting already in utero with high genetic vulnerability. The aim was to study whether offspring of antenatally depressed mothers have an elevated risk for severe mood disorders till middle adulthood, taking account parental severe mental disorder.

**Methods:**

The general population-based Northern Finland 1966 Birth Cohort includes 12,058 children, whose mothers were asked at mid-gestation if they felt depressed. The offspring were followed for over 40 years, and hospitalised severe mental disorders were detected using the Finnish Hospital Discharge Register. It was also used for identifying severe mental disorders in the parents till 1984, when the offspring were of age.

**Results:**

Of the mothers, 14% self-reported depression during pregnancy. Of the parents, 10% had suffered from a hospitalised severe mental disorder.

Adult offspring of antenatally depressed mothers had modestly increased risk for mood disorders both non-psychotic (crude OR 1.6; 95%CI 1.1–2.2) and psychotic (2.0; 1.0–4.1) but not for schizophrenia nor substance use disorder, when compared with the children of mothers without antenatal depression.

Maternal depression during pregnancy combined with parental severe mental disorder increased the risks for severe mental disorders in the offspring widely. The risks for both non-psychotic (crude OR 3.8; 95%CI 2.1–6.6) and psychotic mood disorder (5.4; 1.6–18.1) and also for schizophrenia (4.3; 2.3–8.2) and substance use disorder (2.8; 1.7–4.7) were higher in the offspring with both maternal antenatal depression and parental severe mental disorder than in those without maternal depression and with severe mental disorder in the parent (for non-psychotic 1.5; 1.0–2.4 and psychotic mood disorder 4.2; 1.9–9.2, for schizophrenia 1.3; 0.8–2.4 and for substance disorder 1.5; 1.1–2.2) or in those with a depressed mother but without parental severe mental disorder (for non-psychotic 1.3; 0.9–1.9, and for psychotic mood disorder 2.1; 0.9–5.0, for schizophrenia 0.9; 0.5–1.6 and substance disorder 1.4; 1.1–2.0). The reference group was birth cohort members without maternal antenatal depression and without paren¬tal hospital-treated mental disorder. The risks remained statistically significant even after adjustment for maternal smoking during pregnancy, perinatal complications, father’s social class and family type at birth. In the offspring of antenatally depressed mother and a father with severe mental disorder the risk was elevated only for schizophrenia (7.5; 2.2–26.2).

**Discussion:**

Maternal depression during pregnancy increased the risk for mood disorders in the offspring slightly but not for schizophrenia nor substance use disorder when compared with the children of mothers without antenatal depression. Maternal antenatal depression combined with parental severe mental disorder increased the risks for all of these severe mental disorders in the adult offspring.

The risk was highest for schizophrenia in the offspring of antenatally depressed mother and a father with severe mental disorder.

To our knowledge, this is the first study of mood disorders, schizophrenia and substance use disorder in the offspring of antenatally depressed mothers with long follow-up till middle age where familial vulnerability for severe mental disorders was taken into account in a general population-based sample.

**References:**

1. Mäki P, et al. Am J Psychiatry 2010;167(1):70–7,

2. Taka-EilolaT et al. Eur Psychiatry 2017; 42:36–43

